# Protective Effects of Moderate Ca Supplementation against Cd-Induced Bone Damage under Different Population-Relevant Doses in Young Female Rats

**DOI:** 10.3390/nu11040849

**Published:** 2019-04-15

**Authors:** Xiao Huang, Teng Liu, Meng Zhao, Haowei Fu, Jinming Wang, Qian Xu

**Affiliations:** Key Laboratory of Environmental Medicine Engineering, Ministry of Education, School of Public Health, Southeast University, Nanjing 210009, China; huangxiao1998@163.com (X.H.); liu_teng6016@126.com (T.L.); zhaomengmail@163.com (M.Z.); 220183495 @seu.edu.cn (H.F.); u20130608@163.com (J.W.)

**Keywords:** cadmium, calcium, bone, klotho, fibroblast growth factor 23

## Abstract

Estimation of the skeleton-protective effects of Ca in Cd-induced bone damage is helpful in the assessment of Cd health risk. The aim of this study was to identify whether Ca supplementation during exposure to different population-relevant doses of Cd can prevent Cd-induced bone damage under the tolerable upper intake level of Ca supplementation. Young female Sprague-Dawley rats were given different population-relevant doses of Cd (1, 5, and 50 mg Cd/kg diet) and Ca supplementation (0.4% Ca supplementation) intervention. Ca supplementation significantly decreased Cd-induced bone microstructure damage, increased bone biomechanics (*p* < 0.05), serum bone formation marker level (*p* < 0.05) and expression of osteogenic gene markers exposure to the 5 and 50 mg Cd/kg diets. However, it had no impact on these indicators under the 1 mg Cd/kg diets, with the exception of expression of osteogenic marker genes. Ca supplementation significantly decreased serum Klotho level (*p* < 0.05), and fibroblast growth factor 23/Klotho-associated gene expression in the kidney and bone showed significant changes. In conclusion, Ca supplementation has a positive effect on bone formation and bone quality against the damaging impact of Cd, especially with exposure to the 5 mg and 50 mg Cd/kg diet, which may be related to its impact on the fibroblast growth factor 23/Klotho axis.

## 1. Introduction

Cadmium (Cd) is a ubiquitous environmental contaminant and has a long biological half-life (10–35 years) [1,2,3,4]. Diet is the main route of Cd exposure in the non-smoking general population without occupational exposure [2,5,6]. Prolonged exposure to Cd by inhalation or ingestion can cause kidney damage; Cd accumulates in the kidney and is excreted in the urine as a reflection of the body burden [4]. To prevent kidney damage, the Joint FAO/WHO Expert Committee on Food Additives (JECFA) established a provisional tolerable monthly intake (PTMI) of 25 μg/kg BW/month (58 μg per day for a 70 kg person) and a threshold of urinary Cd of 5.24 mg/g creatinine [7]. There has been growing evidence that Cd has a direct toxic effect on bone and that exposure to this metal via the diet may occur at exposure levels lower than the threshold limits of dietary Cd intake and urinary Cd established by the JECFA [8] Therefore, whether to impose stricter dietary intake guidelines to strengthen health protection against Cd-induced bone injury is a particular concern in Cd health-risk assessment [2,3].

Calcium (Ca) is a confounding factor for Cd-induced bone damage in epidemiological studies [9,10,11]. Ca is one of the major mineral components of the skeletal system. Ca intake is particularly important in acquiring peak bone mass during young adulthood, which lowers risk of bone fracture later in life. With a few exceptions [11,12], few epidemiological studies on the association of low Cd exposure and decreased bone mineral density (BMD), osteoporosis, and increased fracture risk have taken the protective effect of Ca into consideration. Although several studies have adjusted the intake of Ca, it remained between 902–1081 mg [11,12], less than the Ca intake for adults aged 50 years and older (1000–1200 mg) recommended by the American Association of Clinical Endocrinologists and the American College of Endocrinology Clinical Practice Guidelines for the Diagnosis and Treatment of Postmenopausal Osteoporosis—2016 [13]. Furthermore, osteoporosis and fracture caused by Cd exposure is a very long-term process (from birth to old age), and inadequate dietary Ca increases Cd absorption and retention in rats [14,15]. It is therefore not clear whether low Cd exposure or inadequate Ca intake is associated with low BMD and with osteoporosis. Consequently, the skeleton-protective effects of Ca should be considered in the health risk assessment of Cd. If Ca is not taken into account, the Cd health risk will be overestimated.

In experimental animals, the effects of the interactions between Ca and Cd create a complex effect on bone health because different levels of exposure to Cd and Ca have different toxicokinetic characteristics [15,16,17]. However, many previous experimental animal studies have employed Cd doses larger than doses received by humans in the general population (i.e., in unpolluted areas) [17,18,19,20]. In addition, excess Ca in the body is a risk factor for kidney stone formation, impaired absorption of other minerals, and increased cardiovascular events [21,22]. Similarly, previous studies concerning the protective effect of Ca against Cd-induced toxic effects have also used Ca doses higher than the dose considered to be the adequate intake level in humans, and even higher than the tolerable upper intake level [17,18,19]. Also, the intake of the tolerable upper intake level is not a recommended level of intake [17]. Taking the above into account, it seems crucial to consider the risk of potential adverse health effects of Ca supplementation in the antagonism of Cd-induced bone damage. However, there is little toxicological information on the effects of Ca under simultaneous exposure to very-low dietary Cd levels in either humans or experimental animals.

The mechanism under which Ca supplementation protects against the impact of Cd on the skeleton is still poorly understood. Recent data indicate that the fibroblast growth factor 23 (FGF23)–bone–kidney axis is part of a newly-discovered biological system that connects bones to other organ functions through a complex endocrine network that binds to the vitamin D axis, playing an equally important role in health and disease [23,24]. According to Kuro [25], Klotho protein has two forms: membrane Klotho and secreted Klotho. Membrane Klotho functions as an obligate coreceptor for FGF23, and regulates phosphate metabolism and vitamin D synthesis. Soluble Klotho (Klotho) is produced either by alternative splicing of the Klotho gene or by ectodomain shedding on the cell surface by membrane-anchored proteases, and then released into the extracellular fluid. Secreted Klotho functions as a circulating hormone with actions distinct from those of FGF 23. These include regulation of ion channels and transporters and acting as a decoy receptor that disrupts insulin-like growth factor I, Wnt/beta-catenin signaling and transforming growth factor β signaling [24,25]. However, to our knowledge, the involvement of the FGF23/Klotho axis in the pathways of Ca supplementation against Cd-induced bone damage has not been investigated.

Experimental animal models are a powerful tool for nutritional and toxicological studies, since they provide homogenous population samples by controlling dietary and environmental factors, which are difficult to control in human studies. The weanling Sprague–Dawley rat is a useful experimental model to evaluate peak bone mass development because it displays progressive gain in bone parameters during growth [26]. Previous studies have revealed that the female is more susceptible to damage by the heavy metal Cd because of its relatively high absorption rate compared with the male [27,28]. Consequently, female rats were used in this study.

The aim of the present study was to identify whether Ca supplementation can prevent Cd-induced bone damage when exposed to different population-relevant doses, especially exposure to general populations. Results of this study have important implications for Cd health-risk assessment and development of effective dietary strategies to reduce the adverse health effects of Cd exposure.

## 2. Materials and Methods

### 2.1. Animals

One hundred twenty young female Sprague–Dawley rats (65–75 g, four weeks of age) were purchased from Shanghai Jiesijie Experimental Animal Co. Ltd. (license number: SCXK (HU) 2013-0006). All rats were housed in plastic cages in an SPF animal laboratory at Southeast University. The animal laboratory has an animal use certificate issued by the Science & Technology department of Jiangsu Province (SYXK < Jiangsu > 2016-0013). The room was kept at a temperature of 20 ± 0.5 °C and a humidity of 60 ± 10% on a 12-h light–dark schedule. All the rats were given free access to diets and deionized water. The experimental procedures used throughout this study were approved by the Animal Care and Use Committee and the Animal Ethics Committee at Southeast University (approval number: 2015-0623-008).

### 2.2. Experimental Design

#### 2.2.1. Experiment 1

To evaluate the sub-chronic toxicity and lowest observed adverse-effect level (LOAEL) of Ca supplementation with normal dietary Cd levels in the general population, 50 young female Sprague-Dawley rats were given 7 days to acclimatize and were then divided into five groups of 10 rats in a randomized block design based on body weight (Table 1). The control group was fed the AIN-93G diet, which was isocaloric by formulation, and was prepared according to the American Institute of Nutrition Rodent Diets Recommendations for growing animals (AIN-93G) [29]. The AIN-93G diet is also an appropriate diet for safety evaluation toxicity studies in rats, and has a Ca content of 5 g/kg diet (0.5%) [29,30]. With the exception of the control group diet, the Cd_1_ group and all Cd_1_ plus Ca group diets containing 1 mg Cd/kg were prepared by addition of CdCl_2_ (99.996%, total metal impurities, 0.004% max, Alfa Aesar, Shanghai, China) to the ingredients of the standard feed (AIN-93G) [29,30] at the stage of production. The Cd exposure level (1 mg Cd/kg diet) was close to that of the general-population dietary exposure according to previous studies [28]. The lowest dietary Ca supplementation level, 133 mg Ca/kg body weight/day, was equivalent to approximately 10 times the dietary Ca intake of 800 mg/day for a 60 kg individual (13.3 mg/kg BW/day) [31] recommended by the Chinese Dietary Reference Intakes (2013) for optimal nutrition and bone accretion [31]. The maximum dietary Ca level was based on a previous study [32,33], which showed that the amount and total weight of litters decreased when the Ca (CaCO_3_) content in the diet was 1.1%. The intermediate dose was 0.9% Ca content. All three dietary Ca levels were obtained by addition of CaCO_3_ (analytical grade, Xilong Chemical Co., Ltd., Guangdong, China) to the AIN-93G diets. 

The AIN-93G components are listed in Supplementary Table S1. The composition of mineral mixture was based on AIN-93G. It provided (mg/kg diet): Ca, 5000; P, 3000; Mg, 513; Fe, 45; Zn, 38 and Cu, 6. All feed ingredients were combined and thoroughly mixed to ensure homogeneity. The diets were prepared by Trophic Animal Feed High-Tech Co., Ltd. (Nantong, China). According to the Chinese Toxicology Assessment Procedures and Methods for Food Safety (Chinese standard GB15193.13-2015) and previous research [34,35,36], the number of animals required for a 90-day subchronic toxicity study is no less than 10 per group. Therefore, 10 rats were used in our study.

#### 2.2.2. Experiment 2

Based on the LOAEL of Ca supplementation (Exp.1), whether Ca supplementation can prevent Cd-induced bone damage under different population-relevant doses of Cd was investigated. Seventy female rats were randomly divided into seven groups (Table 1). The low (general population [20,28]), moderate (highly-exposed general population [28,37]), and high (polluted areas [38,39]) Cd exposure levels (1, 5, and 50 mg Cd/kg diet) used in this study were very close to human dietary exposure [4,20]. Thereafter, information on the Cd exposures in rats were collected, including the average daily Cd intake, and the Cd content in the kidney, liver, and femur. To evaluate the beneficial effect of Ca supplementation, femoral bone Ca content, bone biomechanics, serum bone formation markers, bone histology and expression of osteogenic marker genes were examined. Finally, serum Klotho and FGF23 levels, and expression of FGF23/Klotho-associated genes in the kidney and bone were examined to explore the mechanism of the FGF23/Klotho axis.

### 2.3. Observations of Rats, Body Weight, and Food Consumption

Mortality, signs of morbidity and clinical signs of toxicity (such as appearance/posture, behavior, feces/urine, body surface, and fluid secretion/excretion) of rats were monitored daily during the feeding trial. Individual body weight and food consumption were also recorded weekly, as described in previous research [34,35,36].

### 2.4. Hematology and Serum Biochemistry

In the 90-day oral toxicity study, before collection of blood samples, rats were subjected to fasting for 16 h with water provided *ad libitum*. The animals were anesthetized with 10% chloral hydrate (3 mL/kg, i.p.) before blood was collected from the inferior vena cava. Approximately 1 mL samples of blood from each animal were collected into blood samplers containing the anticoagulant potassium EDTA-K2. Hematological analysis was performed using a Sysmex XE-2100 total hematology system (Sysmex Corporation, Japan) for hemoglobin (HGB), red blood cell count (RBC), mean corpuscular hemoglobin concentration (MCHC), mean corpuscular hemoglobin (MCH), mean corpuscular volume (MCV), hematocrit (HCT), platelet count (PLT), and white blood cell count (WBC); white blood cell differential count based on their percentages included neutrophils (NEUT), lymphocytes (LYM), monocytes (MONO), eosinophils (EOS), and basophils (BASO).

Blood samples of approximately 3–5 mL from each animal were collected into blood samplers containing no anticoagulant. Biochemical analysis was performed using an AU5800 automated clinical chemistry analyzer (Beckman Coulter, Brea, CA, USA) for aspartate aminotransferase, alanine aminotransferase, creatinine (CRE), blood urea nitrogen (BUN), triglycerides, total cholesterol, glucose (GLU), total protein (TP), albumin, globulin (GLO), albumin/globulin (A/G) ratio, Ca, phosphorus (P), iron (Fe), zinc (Zn), and copper (Cu).

The serum levels of 1,25(OH)_2_D_3_, N-terminal propeptide of type I procollagen, and C-terminal telopeptide fragments of type I collagen were measured using enzyme immunoassay kits (Yifeixue Biotech, Nanjing, China). Serum Klotho levels were determined with the corresponding ELISA kit (Jiancheng Biotech, Nanjing, China) following the manufacturer’s instructions. Serum FGF23 levels were measured using both an intact FGF23 enzyme-linked immunoassay, which exclusively measures the full-length protein, and a C-terminal FGF23 enzyme-linked immunoassay, which recognizes the intact protein and its C-terminal cleavage fragments (Yifeixue Biotech).

### 2.5. Organ Weight and Histopathologic Examination

After collecting the blood samples, rats were killed by cervical dislocation. A thorough necropsy of major organs was performed, and the heart, liver, spleen, and kidneys (paired) were excised, examined, and weighed. Tissue samples were fixed in 4% buffered formaldehyde for more than 24 h before histological processing, and then kidneys were embedded in paraffin in order to create 2–4 μm thick sections, which were stained with hematoxylin–eosin (HE) for light microscopy (Olympus, BX-50, Tokyo, Japan). The renal cortex, medulla, glomerulus, and renal tubules of the kidneys were graded in a blind manner by two independent observers.

In the second experiment, the left tibia was collected and stained with HE following a series of pretreatments as described by Chen [38]. Trabecular number, trabecular thickness, and trabecular separation in the tibia were evaluated.

### 2.6. Determination of the Cd, Fe, Zn, and Cu Content of the Kidneys and Liver

The concentrations of Fe, Zn, and Cu in the kidneys and liver were determined using an atomic absorption spectrophotometer (AA-7000 series; Shimadzu Corp., Kyoto, Japan). Detection was performed in accordance with standard methods (Chinese Standard GB 5009.15-2014 for Cd, Chinese Standard GB 5009.90-2016 for Fe, Chinese Standard GB 5009.14-2017 for Zn, and Chinese Standard GB 5009.13-2017 for Cu).

### 2.7. Bone Biomechanical Testing and Determination of Femoral Bone Ca Content

The left femurs were collected, wrapped in saline-soaked gauze and stored at −20 °C until testing. Each femur was thawed at room temperature and kept wet using 0.9% saline solution before tests. A three-point bending test was performed to evaluate the biomechanical properties of the femoral diaphysis. An Instron 5943 testing machine (Instron Inc., High Wycombe, UK) with a 1 kN loading cell and with a force resolution of 0.05 N was used. During the test, each femur was placed horizontally on two rounded supporting bars located at a distance of 19 mm, with the anterior surface facing upwards [28,40]. The pressing force was applied vertically to the midshaft of the bone. Each bone was compressed at a rate of 1 mm/min until fracture, and the force–displacement data were recorded. All of the tests were performed by the same operator. According to the manufacturer, the measurement error of the method is 0.25% of the recorded value. After the three-point bending test, the vertical and horizontal internal and external heights at the point of the diaphyseal fracture were measured. The cross-sectional area and cross-sectional moment of inertia at the point of the diaphyseal fracture were calculated from the internal and external heights. The yield load, fracture load, and stiffness were used to describe the “structural” properties of the femur as a whole anatomical unit [28,40]. To evaluate the “material” properties (intrinsic; independent of the tissue size) of the bone tissue at the femoral diaphysis, the yield load, fracture load, and stiffness were normalized for their “geometric” properties, to give the yield stress, fracture stress, and elastic modulus (Young’s modulus of elasticity), as described in previous research [28,40].

After biomechanical testing, the femoral bone was collected and dried in an oven at 105 °C to a constant weight, and then the dry weight of the bone was measured using a Mettler–Toledo balance (Mettler–Toledo, Columbus, OH, USA). The concentrations of Ca in femoral bone were determined on an atomic absorption spectrophotometer (AA-7000 series; Shimadzu). Analysis was performed in accordance with the standard method (Chinese Standard GB 5009.92-2016).

### 2.8. Gene Expression

Gene expression of bone differentiation markers and FGF23/Klotho-associated genes in the kidney and right tibia were evaluated by real-time qRT-PCR. Total RNA was isolated from tissues using Trizol reagent (Yifeixue Biotechnology). To isolate total RNA from the femur, we used an RNA extraction kit for bone tissues (Raincarbonnet Biotechnology LLC, Nanjing, China) according to the manufacturer’s protocol. cDNA synthesis was performed using 1 μg of total RNA and a qPCR RT kit (Toyobo, Osaka, Japan). Quantitative real-time polymerase chain reaction was performed on a thermal cycler and a real-time system (Bio-Rad Laboratories, Hercules, USA) using a SYBR Green qPCR Master Mix (Vazyme, Nanjing, China). GAPDH served as the reference gene to normalize expression. The specific primer sets for the target gene used are listed in Supplementary Table S2.

### 2.9. Statistical Analyses

Data are represented as mean ± SD. Data within groups were initially analyzed using Levene’s test for homogeneity of variances and the Shapiro–Wilk test for normality. If variances were considered to be not significantly different, then statistical significance between groups was determined using one-way ANOVA. *Post hoc* tests were executed by Dunnett’s test for comparisons against control values. Where variances were considered significantly different by Levene’s test, groups were compared using a non-parametric method (Kruskal–Wallis non-parametric analysis of variance followed by Dunn’s test). Chi-square, Kruskal–Wallis or Fisher’s exact test were used for incidence data. Differences among groups were judged to be statistically significant at a probability of *p* < 0.05. All statistical analyses were performed using Statistical Package for Social Sciences software, version 17.0 (SPSS Inc., Chicago, IL, USA).

## 3. Results

### 3.1. LOAEL of Ca supplementation with dietary exposure to 1 mg Cd/Kg (Exp. 1)

#### 3.1.1. General effects, body weights, food consumption, and Cd intake

There were no deaths during the administration period in any of the study groups. No signs of toxicity were observed in any of the groups. There were no significant differences in body weight (Table 2) between rats fed different diets. Mean weekly feed consumption is shown in Supplementary Table S3. From the daily food consumption, we calculated the total food consumption and mean feed efficiency (Table 3). No food consumption or food efficiency changes were attributable to high-level dietary Ca administration. Any changes were considered incidental and without biological or toxicological significance.

The average daily Cd intake, calculated based on feed consumption, ranged from 55.2 to 75.0 μg/kg/BW/day, as shown in Table 3. There was no significant difference in average daily Cd intake between the Cd_1_ control group and the different Cd_1_ plus Ca groups.

#### 3.1.2. Hematology

A summary of the hematological parameters is presented in Table 4. Significant increases, or a tendency thereto, in WBC, MONO, and LYM were noted for the Cd_1_ + Ca_H_ group. Some statistically significant differences were observed between the control and Cd_1_ + Ca groups; however, none was considered Ca-related because of a lack of a clear dose response. These differences included higher NEUT in the Cd control group and EOS in the Cd_1_ + Ca_L_ group.

#### 3.1.3. Serum Chemistry

A summary of the serum chemistry parameters is presented in Table 5. Significant increases, or a tendency thereto, in BUN was noted in the Cd_1_ + Ca_M_ and Cd_1_ + Ca_H_ groups (Table 5). Decreases were observed in total protein in the Cd_1_ + Ca_M_ group, globulin in the Cd_1_ + Ca_L_, Cd_1_ + Ca_M_, and Cd_1_+Ca_H_ groups, and these results were not considered to be Ca-related because of a lack of dose dependency.

#### 3.1.4. Organ Weights and Kidney Histopathology

**Figure 1:**
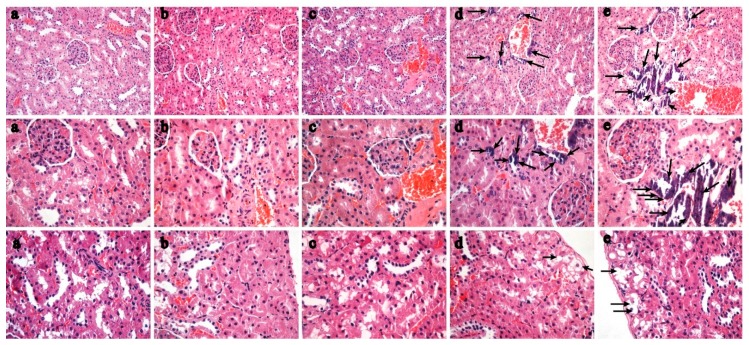
Representative HE images of kidney in female rats fed the AIN93G diet without Cd (control) (**a**), with 1 mg Cd/kg diet (**b**), and with 1 mg Cd/kg diet plus 0.15% Ca supplementation (**c**), with 1 mg Cd/kg diet plus 0.4% Ca supplementation (**d**), with 1 mg Cd/kg diet plus 0.6% Ca supplementation Ca (**e**). Tubular epithelial cell calcification and tubular vacuolation are shown in (**d**,**e**).

No Ca-related adverse gross lesions were observed during the entire necropsy process. No statistically significant difference was observed in absolute organ weight or relative organ weight between groups (Supplementary Table S4). Microscopic findings by HE staining in the kidneys which were considered to be Ca-related were as follows: Increased diffuse tubular epithelial cell calcification in the proximal tubules (severity scale from minimal to moderate) of the Cd_1_+Ca_M_ group and the Cd_1_+Ca_H_ group (Figure 1), with higher grade and incidence than in the normal control and Cd_1_ control. Tubular vacuolation was observed in the kidneys in the Cd_1_+Ca_M_ group and the Cd_1_ + Ca_H_ group. Other findings observed in Ca-related groups were considered to be those found in historical controls or to be incidental because of their low incidence and/or characteristics (Supplementary Table S5).

#### 3.1.5. Fe, Zn, Cu, Cd content of the Kidney and Liver and Ca Concentration in Femur

Significant decreases, or a tendency thereto, in Fe were noted in the liver and kidneys in the Cd_1_+Ca_H_ group. The trends of Fe concentration in the liver were similar to those in the kidney cortex (Table 6). In addition, the Cd concentration in the kidney cortex in Cd_1_ control and all Cd plus Ca groups ranged from 1.21 to 5.28 μg/kg. The maximum Cd concentration in the kidney cortex was 5.28 μg/g, which is lower than the generally accepted lower limit (50–200 μg/g wet weight) that is considered to be safe for the general population [2,41]. The femoral bone Ca content with dietary exposure to 1 mg Cd/kg showed no significant difference with increasing dietary Ca levels. However, there was a decreasing trend in the Cd_1_+Ca_H_ group (Table 6).

### 3.2. Cd intake and Cd Concentration in the Kidney, Liver and Femur Following Exposure to Different Levels of Dietary Cd (Exp. 2)

The body weight of all rats increased throughout experiment 2 (Table 7). No significant differences in body weight were found in rats treated with Cd compared with the normal controls. In addition, no significant difference of body weight was observed in rats treated with Cd with or without Ca supplementation.

During the whole of experiment 2, the Cd intakes from the 1, 5, and 50 mg Cd/kg diets were 56–90 μg/body weight/day, 325–510 μg/body weight/day, and 3800–5300 μg/body weight/day, respectively. No significant differences in Cd intake were observed between rats treated with or without Ca supplementation.

Cd concentrations in the kidney (Table 8), liver, and femur of normal control rats (without Cd) were at very low levels, below the detection limits. Exposure to Cd resulted in a dose-dependent increase in the body burden of this metal. Cd levels in kidney, liver, and femur were much higher in the Cd exposure groups compared with controls, in particular, those treated with the 5 and 50 mg Cd/kg diets (*p* < 0.01). However, no significant differences in Cd concentrations in the kidney, liver, or femur were observed in rats treated with Cd with or without Ca supplementation.

### 3.3. Protective Effect of Ca Supplementation with Dietary Exposure to Different Levels of Cd (Exp. 2)

#### 3.3.1. Alterations in Bone Histomorphometry and Femoral Bone Ca Content

**Figure 2:**
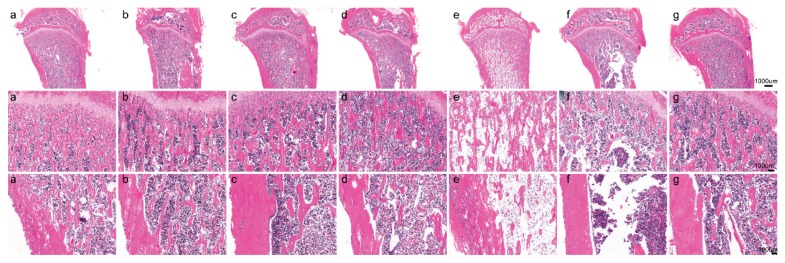
Representative HE staining of distal left tibia sections without Cd (**a**); with 1 mg Cd/kg diet Cd (**b**), with 1 mg Cd/kg diet plus Ca (**c**), 5 mg Cd/kg diet (**d**), 5 mg Cd/kg diet plus Ca (**e**), 50 mg Cd/kg diet (**f**), and 50 mg Cd/kg diet plus Ca (**g**). The trabecular number and cortical thickness decreased and that the trabecular separation increased in d, f groups. Ca supplementation led to a significantly increased in trabecular number and cortical thickness (**e**,**g**). But there were no significant differences in exposure to the 1 mg Cd/kg diet with (**c**) or without Ca supplementation (**b**).

The results presented in Figure 2 show the protective effect of Ca supplementation on bone with exposure to different diets containing Cd. HE staining of the tibia revealed that the trabecular number and cortical thickness decreased and trabecular separation increased in Cd exposure groups, especially the 50 mg Cd/kg diet group, relative to the control group (Figure 2f). Exposure to the 5 and 50 mg Cd/kg diet with Ca supplementation led to a significant increase in trabecular number and cortical thickness (Figure 2d–g), but there were no significant differences with exposure to the 1 mg Cd/kg diet with or without Ca supplementation (Figure 2a–c).

No significant differences were observed in left femur dry weight between rats treated with Cd alone or with Cd plus Ca compared with the controls (Supplementary Table S6). With exposure to the 50 mg Cd/kg diet, Cd caused a significant decrease in the femoral bone Ca content relative to the control (Table 9). Ca supplementation led to a significant increase in femoral bone Ca content with exposure to the 50 mg Cd/kg diet, but there were no significant differences with exposure to the 1 and 5 mg Cd/kg diets with or without Ca supplementation (Table 9). In addition, the femoral bone Ca content with dietary exposure to 1 mg Cd/kg (Exp. 1) showed no significant difference with increasing dietary Ca levels. However, there was a decreasing trend in the high Ca supplementation group (Table 6).

#### 3.3.2. Effects on Bone Strength

The mechanical properties of bone are shown in Table 10. Exposure to dietary Cd at 5 and 50 mg Cd/kg caused a significant decrease in Young’s modulus (MPa) and fracture stress, relative to the control (without Cd), whereas exposure to 1 mg Cd/kg had no statistically significant effects (Table 10). Ca supplementation led to significant increases in Young’s modulus (MPa) and fracture stress in rats exposed to 5 and 50 mg Cd/kg in the diet, but there were no significant differences with exposure to the 1 mg Cd/kg diet with or without Ca supplementation (Table 10).

#### 3.3.3. Effects on Serum Markers and Osteogenesis

Consistent with the data regarding bone mechanical properties, Ca supplementation led to a significant increase in the bone formation marker N-terminal propeptide of type I procollagen in rats exposed to 5 and 50 mg Cd/kg, but there were no significant differences with exposure to the 1 mg Cd/kg diet with or without Ca supplementation (Table 9). Ca supplementation did not change levels of the bone resorption marker C-terminal telopeptide fragments of type I collagen in rats exposed to dietary Cd at 1 and 5 mg Cd/kg, but it significantly increased with the 50 mg Cd/kg diet (Table 9). Quantitative real-time polymerase chain reaction analysis also confirmed that Ca supplementation resulted in upregulation of osteogenic marker genes, including dentin matrix protein 1 and collagen type 1 in the 1 mg and 50 mg Cd/kg diet groups (Table 11). These results indicate that Ca supplementation increased osteoblast activity, which in turn contributed to increases in bone formation and bone quality.

### 3.4. Serum Calciotropic Hormones and FGF23/Klotho-Associated Gene Expression in the Kidney and Bone (Exp. 2)

#### 3.4.1. Serum Calciotropic Hormones

The results presented in Table 12, Table 13 and Table 14 show the levels of serum calciotropic hormones and FGF23/Klotho-associated gene expression in kidney and bone. We focused on these changes, especially in the high-Cd-exposure group, in order to explore the mechanisms of how Ca supplementation protects against the impact of Cd on the skeleton. Serum levels of Klotho were significantly decreased in the Ca supplementation group compared to the group without Ca supplementation with dietary exposure to 1, 5, and 50 mg Cd/kg (Table 12). A slight increase in 1,25(OH)_2_D_3_, intact FGF23 and C-terminal FGF23 was found in rats treated with Ca supplementation compared with Cd alone (without Ca supplementation), but the differences were not significant (Table 12 and Figure S1).

#### 3.4.2. FGF23/Klotho-Associated Gene Expression in the Kidney

In accordance with these biochemical results, gene expression of Klotho in the kidney also decreased in rats treated with Cd plus Ca compared with rats treated with Cd alone in the 1, 5, and 50 mg Cd/kg diet groups (Table 13). Moreover, gene expression of Fgfr1 significantly increased with exposure to the 5 and 50 mg Cd/kg diets. Gene expression of Napi2a significantly decreased in rats treated with Cd plus Ca compared with rats treated with Cd alone with low, moderate, and high Cd exposures. There was a significant increase in the expression of Cyp27b1 in rats exposed to Cd plus Ca compared with rats treated with Cd alone when fed the 1 and 50 mg Cd/kg diets. The gene expression of Cyp24a1 showed a tendency to increase in rats treated with high level Cd plus Ca compared with those treated with high level Cd alone, but this was not statistically significant (Table 13). These results indicate that Klotho/FGF23 in the kidney has an impact on regulation of mineral metabolism in our rat model of human exposure and that it is involved in the mechanisms of how Ca protects against the impact of Cd on the skeleton.

#### 3.4.3. FGF23/Klotho-Associated Gene Expression in Bone

A significant increase in FGF23 expression in bone was observed in rats treated with Cd alone and with Cd plus Ca compared with those without Cd (control) (Table 14). Rats receiving Ca supplementation exhibited a significant increase in FGF23 expression when exposed to 1, 5, and 50 mg Cd/kg diets (Table 14). The Ca-supplementation rats in the 1, 5, and 50 mg Cd/kg diet groups displayed a significant increase in OPG expression, whereas there was no change in RANKL expression, which is a marker of osteoclastogenesis.

Increased expression of the Wnt target genes Tcf1 and Axin2 was observed in rats treated with Cd plus Ca compared with those treated with Cd alone in the 50 mg Cd/kg diet group (Table 14). An increase in expression of the Wnt pathway inhibitor Dickkopf-1 (Dkk1) was also observed in the Cd plus Ca group compared with Cd alone in the 50 mg Cd/kg diet group. In terms of Wnt1 and β-catenin, there was a trend towards decreased gene expression with Cd and Ca co-exposure compared with Cd alone, but this was not statistically significant. Taken together, these results (Table 14) suggest that the skeletal effect of Ca supplementation in protecting against the damaging impact of Cd may be related to its impact on FGF23, OPG/RANKL, and Wnt signaling in femurs.

## 4. Discussion

In the daily diet, Cd rarely exists in isolation but rather occurs in the context of numerous other protective substances (such as Ca) [17,19,20,42]. Most epidemiological studies and animal models of Cd toxicity have examined the effects of Cd as an independent variable, rather than in the presence of Ca, an important element antagonist of Cd-induced toxic effects [3,8,9]. Furthermore, excessive Ca consumption may have potential adverse effects on health [21,22]. In the present study, the sub-chronic toxicity of Ca intake combined with exposure to dietary Cd in the general population was evaluated using a rat model. In addition, the question of whether Ca supplementation during low-level (general populations), moderate (highly-exposed general population), and high (polluted areas) sub-chronic exposure to Cd can prevent Cd-induced bone damage under a tolerable upper intake level of Ca supplementation was also investigated. The involvement of the FGF23/Klotho axis was also investigated to explore the mechanism through which Ca protects against the impact of Cd on the skeleton.

### 4.1. LOAEL of Ca Supplementation with Exposure to Cd Under General Population-Relevant Doses in Young Female Rats

It is well known that for a chemical or food additive, the no observed adverse effect level (NOAEL) or LOAEL, which is primarily derived from repeated dose toxicity studies in rodents, is a critical basis for the establishment of a health-based guidance value, a critical value for protecting people from potential adverse health effects [35,36]. Many international organizations have designed various guidelines to assess the safety of chemical or food additive products, such as the Food and Agriculture Organization (FAO) of the United Nations, the World Health Organization (WHO), the Organization for Economic Co-operation and Development (OECD), and the Codex Alimentarius Commission (CAC) [43]. Regardless of country-of-origin or governing organization, a 90-day oral toxicity study is traditionally used to assess the safety of a chemical or food additive product [34,35,36]. In this study, a 90-day oral toxicity study was conducted and the sub-chronic toxicity and the LOAEL of Ca supplementation combined with exposure to normal dietary Cd levels in the general population were evaluated.

The results showed that the rat model of exposure to a 1 mg Cd/kg diet used in the 90-day oral toxicity study can reflect dietary Cd exposure in the general population (in unpolluted areas). First, the mean daily dose of Cd intake in the Cd_1_ group and all Cd_1_ plus Ca groups was 55.2 to 75.0 μg/kg BW/day. These doses are generally recognized as safe, being less than 100-fold lower than the provisional tolerable monthly intake (PTMI) [3]. Second, a previous study conducted by Brzóska et al. [28] supported the theory that low-level exposure to a 1 mg Cd/kg diet in a rat model can reflect the exposure of humans inhabiting an unpolluted area, because the non-smoking adult population has urinary Cd concentrations close to 0.5 μg/g creatinine [20] and the urine Cd concentration in a rat model of lifetime low-level exposure to a 1 mg Cd/kg diet was 0.107–0.285 µg/g creatinine after 17–24 months [28]. Third, the kidney-critical Cd concentration supported the hypothesis that the rat model can reflect dietary Cd exposure in the general population, because human exposure to Cd is assumed to damage the kidneys, especially the proximal tubular cells. Tubule dysfunction may occur if the Cd concentrations in the kidney cortex rise above a critical threshold concentration (50–200 μg/g wet weight) [2,41]. The highest kidney-critical concentration of Cd in all Cd exposure groups (except the control group) was 5.28 μg/g, which is lower than the generally accepted toxic lower limit that is considered safe for the general population. Finally, histopathology and serum chemistry results also supported the above conclusion. There were no significant differences between the Cd_1_ exposure group and the normal control group. These results collectively indicate that the rat model used in our 90-day oral toxicity study can reflect dietary Cd exposure in the general population, and adverse effects found in the 90-day oral toxicity study were not attributable to Cd administration but instead were attributable to high-level dietary Ca administration.

The results from our 90-day oral toxicity study showed that there were no deaths or changes in body weight, body weight gain, food consumption, or food efficiency attributed to Ca administration. In the kidney, significant increases, or a tendency thereto, in tubular epithelial cell calcification and tubular vacuolation were seen in the Cd_1_+Ca_M_ and Cd_1_+Ca_H_ groups, together with significant increases, or a tendency thereto, in BUN in the same groups. These changes were considered to be attributable to high-level dietary Ca administration. The kidneys are the major organ of Ca excretion. Approximately 8000–10,000 mg Ca/day is filtered at the glomerulus, and approximately 65% of Ca is reabsorbed by the proximal tubule [33,44]. Extensive calcification of renal convoluted tubular cells and the tubular lamina have been described in kidney biopsies of patients with milk-alkali syndrome or calcium–alkali syndrome; the extent of Ca deposition was proportional to renal dysfunction [33,44]. Renal dysfunction can lead to mineral and bone disorder. In the Cd_1_+Ca_H_ group, there was a trend toward a decrease in the femoral bone Ca content. Additionally, significant decreases, or a tendency thereto, in iron were seen in the liver and kidneys of the Cd_1_ + Ca_H_ group. These changes were considered to be related to high-level dietary Ca, which reduces Fe absorption, resulting in decreased Fe storage in the liver and kidneys. Based on the principles of the product safety assessment and the above-mentioned results, the LOAEL of Ca exposure in the context of a 1 mg Cd/kg diet in this 90-day oral toxicity study was estimated to be below the Ca concentration in the Cd_1_+Ca_M_ group (0.9% Ca) for female rats. Notably, current findings on the LOAEL of Ca with dietary exposure to 1 mg Cd/kg are lower relative to those for Ca alone (1.1%) [32,44].

### 4.2. Protective Effects of Moderate Ca Supplementation against Cd-Induced Bone Damage Under Different Population-Relevant Doses in Young Female Rats

Based on the above results, the LOAEL of supplementary Ca (moderate Ca supplementation) was used as the tolerable upper intake level of Ca supplementation to determine whether Ca supplementation has skeleton-protective effects under different levels of Cd in actual dietary exposure. To our knowledge, this study is the first report of an experimental study on Ca supplementation effects in the presence of low-level sub-chronic exposure to Cd. Compared with the Cd alone group, Ca supplementation demonstrated a pivotal protective role in the regulation of bone formation and bone quality. Ca supplementation with dietary exposure to 50 mg Cd/kg (polluted areas relevant dose) revealed that exposure to Cd protected against heavy-metal-induced disorders of femoral bone Ca content, bone biomechanics, serum bone formation markers, bone histology, and bone gene expression. With dietary exposure to 5 mg Cd/kg (highly-exposed general population relevant dose), the Ca-supplemented group showed beneficial effects on bone biomechanics, serum bone formation marker levels, bone histology, and bone gene expression compared with the Cd group, but there was no impact on the femoral bone Ca content. At dietary exposure levels of 1 mg Cd/kg (general populations relevant dose), however, Ca only exhibited a partial protective effect on femoral gene expression of osteogenic markers, but had no impact on bone biomechanics, serum bone formation markers, or bone histology. These results collectively indicate that Ca supplementation has a positive effect on bone formation and bone quality in protecting against Cd-induced bone damage. Moreover, the beneficial effect of Ca supplementation was dependent on the Cd dose. Although we could not detect an increase in bone biomechanical properties with exposure to the 1 mg Cd/kg diet, we cannot exclude this possibility because Ca intake plays an important role in the proper growth, development, and maintenance of healthy bones [13]. Cd accumulates in osteocytes, the periosteum, and bone marrow but not in hydroxyapatite [8]. Cd exposure directly inhibits Ca incorporation into osteoid at the mineralization front [45]. It has also been suggested that Ca supplementation has the capacity to regulate the perilacunar matrix, enhance bone mineralization during growth, decrease bone loss and reduce the risk of osteoporotic fracture in the elderly [46]. In our study, the increase in femoral gene expression of osteogenic markers, including collagen type 1, runt-domain transcription factor 2, and matrix metalloproteinase 1 support this possibility. However, it should be acknowledged that the increased expression of osteogenic marker genes could not have provided protection against Cd-induced bone damage (increased risk of low BMD, osteoporosis, and fractures) in the future because the experiment lasted 90 days and could not reflect the protective effect of lifetime Cd exposure. The exact effect of Ca antagonism of Cd-induced toxic effects on bone with exposure to the 1 mg Cd/kg diet (general population dose) remains unclear, and further investigation is necessary.

### 4.3. Exploration of Potential Mechanisms Underlying the Effect of Ca in Protecting against Cd-Induced Bone Damage via the FGF23/Klotho Axis

Cd is a non-essential metal absorbed through one or more transporters of essential metal ions (Zn, Mg, Ca, and Fe) [16,47,48]. Interactions between Ca and Cd may occur at various stages of metabolism of both metals, i.e., absorption, distribution in the organism, and excretion [15,16]. Deficiency of some bioelements can increase the gastrointestinal absorption and accumulation of Cd in the organism, while their enhanced intake may prevent absorption of this toxic element and reduce accumulation of this metal in the organism, ultimately leading to a reduction in the toxic effects of Cd [4,15,47]. Decreased Cd accumulation in the tissue is a well-known mechanism via which Ca protects against this impact [15,17]. In contrast to the Cd group, the Ca supplementation group had no impact on reducing Cd accumulation in the tissue, but it resulted in a marked increase in bone formation and bone quality, along with the enhanced expression of osteoblastic marker genes. There may be different mechanisms through which Ca protects against Cd-induced bone damage.

Currently, the mechanism of Cd osteotoxicity remains unclear. In general, it is considered that the osteopathy is triggered by a renal dysfunction (indirect mechanism) or caused by direct effects of Cd on bone tissues (direct mechanism) [45,46,49]. Potential direct mechanisms of Cd toxicity may include disturbing Ca metabolism and calciotropic hormones; alteration of collagen matrix; stimulation of osteoclast proliferation and activity; decreases in osteoblast viability, mineralization capacity, and alkaline phosphatase activity; increased serum levels of parathyroid hormone and up-regulation of RANK [46,49]. In addition, Cd also affects homeostasis of other minerals involved in bone metabolism. A previous study found that liver levels of iron, magnesium, and selenium decreased while copper, zinc, and manganese increased with increasing Cd levels [46,50]. The current understanding is that kidney effects are important in high-Cd exposure situations, and the osteoporosis that is observed at low Cd exposure levels may be independent of kidney effects [8]. In the present study, Ca supplementation significantly reduced the serum Klotho level in rats, regardless of Cd dosage. This finding suggests that Klotho is involved in the mechanisms of the protective action of Ca on bone tissue.

Cd can directly affect the activity of osteoblasts and osteoclasts, resulting in an imbalance between bone resorption and formation [46,49]. A previous review showed that Klotho has both FGF23-dependent and independent effects on bone [51]. It can act as a decoy receptor that disrupts Wnt/beta-catenin signaling [51,52]. The Wnt/β-catenin pathway is one of the key pathways through which osteocytes regulate osteoblast activity [53,54]. In the presented study, increased expression of the Wnt target genes Tcf1 and Axin2 was observed with changes in Klotho. A previous study identified Klotho as a Wnt inhibitor and demonstrated augmented Wnt biological activity in the proximal tibia of 2-week-old kl/kl mice [55]. Thus, the results suggest that decreased Klotho results in activation of the osteoblastic Wnt pathway, thereby increasing bone formation. Although we were unable to detect decreased expression of Wnt antagonists such as sclerostin and Dkk1 [56], which inhibit osteoblast differentiation and bone formation, one possible reason is that the Wnt/β-catenin pathway may have a more profound impact on bone formation during earlier growth periods under different Cd exposures. The exact mechanism by which Klotho regulates bone formation and mineralization remains unclear, and further investigation is necessary.

Accumulation of Cd within the renal cortex has been proposed to indirectly and directly interfere with enzymes involved in 1,25(OH)_2_D_3_ production and with transporters involved in calcium and Pi homeostasis [49,57]. The principal role of Klotho is to form a specific receptor complex with fibroblast growth factor (Fgf) receptor 1 (Fgfr1) through which it mediates the biological function of FGF23 [24,52]. The main functions of FGF23 are its systemic effects, acting as a counter-regulatory hormone for 1,25(OH)_2_D and coordinating renal phosphate handling to match bone mineralization [24,52]. In the presented study, gene expression of Klotho in the kidney decreased while gene expression of Fgfr1 significantly increased and that of Cyp27b1 increased with exposure to a high Cd diet. In the kidney, FGF23–Klotho signaling inhibits renal phosphate reabsorption by internalizing the sodium-dependent phosphate cotransporters Napi2a and Napi2c, as well as suppressing 1,25-dihydroxyvitamin D [1,25(OH)_2_D_3_] synthesis by altering the vitamin D metabolizing enzymes CYP27b1 and CYP24a1. Recent studies have shown that there is a Ca–FGF-23 endocrine loop, whereby Ca stimulates FGF-23 in bone and FGF-23 stimulates Ca reabsorption in the distal tubule [52,58]. Thus, the findings of this study suggest that Ca stimulates FGF-23 in bone, FGF-23 targets FGFR/a-Klotho complexes in both the proximal and distal tubule, and the effects of FGF-23 in the kidney are inhibited by Npt2a and Npt2c sodium-dependent phosphate co-transporters. These effects result in a decrease in the renal phosphate match to bone mineralization and upregulation of Cyp27b1, resulting in a slight increase in serum 1,25(OH)_2_D_3_.

Cd exposure may inhibit the production of 1, 25(OH)_2_D, which subsequently diminishes calcium uptake in the intestine, resulting in an imbalance between bone resorption and formation [46,49]. In the present study, the slight increase in vitamin D may be involved in the increase in bone formation and bone quality. A recent observation has shown that in osteoblasts and osteocytes, 1,25(OH)_2_D_3_ upregulates FGF-23 expression by binding to the vitamin D receptor (VDR) that forms a heterodimer with retinoid X receptor (RXR), thereby modulating vitamin D response elements (VDREs) in the FGF-23 gene [58,59]. 1,25(OH)_2_D_3_ also indirectly regulates FGF-23 expression through other signaling proteins involving the induction of STAT1, STAT3, and ETS1 [59,60]. In addition, there is an FGF-23 vitamin D counter-regulatory loop, whereby 1,25(OH)_2_D stimulates FGF-23 and FGF-23 suppresses 1,25 (OH)_2_D_3_ levels by inhibiting Cyp27B1 and by stimulating Cyp24A1 in the renal proximal tubule [52,58,60].

Taking into account the above findings, this mechanism is a physiological compensation mechanism which responds to Cd administered under special conditions (the LOAEL of Ca supplementation, young female rats, and exposure to the 50 mg Cd/kg diet). This emphasizes the need for caution when interpreting data. First, only female rats were used, and Cd has been discussed as an endocrine disruptor. Some studies have shown that Cd can bind to the estrogen receptor, that sex hormones influence Cd metabolism and toxicity, and that the female skeleton is susceptible to the damaging impact of this heavy metal [27,61,62]. Second, the protective effect of dietary Ca on Cd-induced bone damage is complex and depends on Ca intake and on the level of Cd exposure [15,20]. Third, age and life stage should also be fully taken into consideration [4,14]. Finally, parathyroid hormone (PTH) also increases calcium efflux from bone [24,25]. Cd interferes with PTH stimulation of vitamin D activation in kidney cells [50]. Klotho is also expressed in the parathyroid gland, where FGF23–Klotho signaling inhibits the synthesis and secretion of PTH [24]. Thus, the action of Klotho may be mediated via the PTH pathway. The exact mechanism by which Ca supplementation protects against the impact of Cd on the skeleton remains unclear. However, our results provide new and important data regarding the protective influence of Ca on the skeleton under sub-chronic exposure to Cd. It seems that the attention of researchers should be drawn to the possibility of using agents mediating the FGF23/Klotho axis in the protection and treatment of Cd-induced bone damage.

### 4.4. Limitations

First, the LOAEL of Ca supplementation (moderate Ca supplementation) was used as the tolerable upper intake Ca supplementation level to determine whether Ca supplementation has skeleton-protective effects under different levels of Cd in actual dietary exposure. However, the Ca intake at the tolerable upper intake level is not a recommended level of intake. There is a risk of both overestimating the protective effects of Ca and underestimating its adverse effects. Further research and risk-benefit analysis should be undertaken to prevent adverse effects from arising from inappropriate advice. Second, the data are from animal studies, and there are major differences between animal species and humans and between different human individuals with regard to the fate of foreign compounds in the body. Furthermore, the factors determining the absorption and accumulation of the ingested Cd include the duration of exposure, dose, and chemical form of the metal, as well as age, sex, diet, and health condition of an exposed person, which should all be taken into account in the estimation of the skeleton-protective effects of Ca in Cd-induced bone damage in human.

## 5. Conclusions

The findings presented here suggest that Ca supplementation has a positive effect on bone formation and bone quality in ameliorating the damaging impact of Cd, especially with dietary exposure to 5 mg and 50 mg Cd/kg. The protective effect of Ca supplementation against the damaging impact of Cd on the skeleton may be related to its impact on the FGF23/Klotho axis, including down-regulation of Klotho mediation of the Wnt/β-catenin pathway in osteoblasts and the function of Klotho as a specific receptor complex with FGF23 in vitamin D synthesis in the kidney and as a coordinator of renal phosphate handling to match bone mineralization.

## Figures and Tables

**Table 1:** Experimental design.

Group.	Diet	Dietary Ca Supplementation, %	Cd (mg/kg)	N	Body Weight	*p*
Control ^a^	AIN-93	0	0	10	132.9 ± 12.92	0.552
Cd_1_	AIN-93+Cd_1_	0	1	10	134.2 ± 16.74	
Cd_1_+Ca_L_	AIN-93+Cd_1_+Ca_L_	0.15% Ca	1	10	140.4 ± 17.16	
Cd_1_+Ca_M_	AIN-93+ Cd_1_+Ca_M_	0.4% Ca	1	10	142.6 ± 11.98	
Cd_1_+Ca_H_	AIN-93+ Cd_1_+Ca_H_	0.6% Ca	1	10	138.7 ± 14.70	
Control ^a^	AIN-93	0	0	10	127.9 ± 12.92	0.570
Cd_1_	AIN-93+Cd_1_	0	1	10	129.2 ± 16.75	
Cd_1_+Ca_M_	AIN-93+ Cd_1_+Ca_M_	0.4% Ca	1	10	137.6 ± 11.98	
Cd_5_	AIN-93+Cd_5_	0	5	10	132.8 ± 14.46	
Cd_5_+Ca_M_	AIN-93+ Cd_5_+Ca_M_	0.4% Ca	5	10	129.9 ± 12.96	
Cd_50_	AIN-93+Cd_50_	0	50	10	131.5 ± 14.78	
Cd_50_+Ca_M_	AIN-93+ Cd_50_+Ca_M_	0.4% Ca	50	10	134.9 ± 14.67	

^a^ The control group fed the AIN93G diet. The AIN-93G diet was prepared according to the American Institute of Nutrition Rodent Diets Recommendations for growing animals and the Ca content in the AIN-93G diet was 5 g/kg diet (0.5%) [29]. The diets contain 1 IU Vitamin D_3_ per 1 g diet. The AIN-93G components are listed in Supplementary Table S1. The mean Ca and Cd concentrations in the diets were determined by atomic absorption spectrometry.

**Table 2:** Body weight of female rats in the 90-day oral toxicity study of Ca supplementation with dietary exposure to 1 mg Cd/Kg (Exp. 1).

Week	Control	Cd_1_	Cd_1_+Ca_L_	Cd_1_+Ca_M_	Cd_1_+Ca_H_	*p*
0	132.90 ± 12.92	134.20 ± 16.75	140.40 ± 17.17	142.60 ± 11.98	138.70 ± 14.70	0.552
1	159.20 ± 16.54	160.60 ± 21.98	163.80 ± 19.95	163.00 ± 13.19	162.30 ± 16.10	0.979
2	177.40 ± 18.58	178.40 ± 21.34	182.90 ± 20.86	178.70 ± 16.93	182.30 ± 18.31	0.955
3	198.70 ± 21.36	203.20 ± 27.07	202.20 ± 23.11	200.20 ± 18.62	205.80 ± 19.36	0.960
4	236.90 ± 33.38	244.10 ± 38.19	234.80 ± 24.80	232.40 ± 18.59	241.70 ± 21.34	0.880
5	267.00 ± 42.05	274.60 ± 50.24	258.50 ± 27.04	254.80 ± 24.40	270.40 ± 27.10	0.711
6	282.40 ± 43.94	295.40 ± 55.73	275.90 ± 26.91	271.00 ± 23.76	286.00 ± 29.79	0.655
7	287.10 ± 47.27	304.20 ± 61.41	288.20 ± 27.74	279.40 ± 23.01	295.60 ± 31.21	0.712
8	294.00 ± 45.44	314.00 ± 70.81	286.20 ± 27.73	282.70 ± 28.93	299.00 ± 32.17	0.544
9	306.40 ± 48.40	335.80 ± 75.48	304.60 ± 28.44	296.40 ± 27.26	312.30 ± 32.83	0.395
10	311.20 ± 49.64	343.60 ± 82.78	309.40 ± 26.12	299.30 ± 26.22	315.50 ± 32.34	0.332
11	322.00 ± 53.04	359.20 ± 94.78	320.80 ± 27.10	311.10 ± 27.21	325.90 ± 33.46	0.335
12	333.40 ± 54.54	355.70 ± 87.49	327.10 ± 26.46	315.60 ± 28.05	331.50 ± 31.89	0.524
13	332.50 ± 52.83	363.30 ± 91.43	337.20 ± 27.46	325.70 ± 26.13	341.40 ± 31.10	0.563

Data are presented as mean ± SD (n = 10). The Control, Cd_1_, Cd_1_+Ca_L_, Cd_1_+Ca_M_, and Cd_1_+Ca_H_ groups respectively represent animals fed the AIN93G diet without Cd, with 1 mg Cd/kg, with 1 mg Cd/kg plus 0.15% Ca supplementation, with 1 mg Cd/kg plus 0.4% Ca supplementation, and with 1 mg Cd/kg plus 0.6% Ca supplementation.

**Table 3:** Total weight gain, total food consumption, mean feed efficiency, and daily Cd intake of female rats in the 90-day oral toxicity study of Ca supplementation with exposure to 1 mg Cd/Kg (Exp. 1).

	The Total Gain of Weight (g)	The Total Food Consumption (g)	Mean Feed Efficiency ^a^ (%)	Daily Cd Intake (μg/BW/day)
Control	209.3 ± 38.59	1592.36 ± 94.73	0.13 ± 0.02	——
Cd_1_	227.4 ± 82.1	1637.98 ± 98.57	0.14 ± 0.04	62.64 ± 9.66
Cd_1_+Ca_L_	197.8 ± 20.38	1550.45 ± 47.04	0.13 ± 0.01	62.47 ± 5.81
Cd_1_+Ca_M_	176.2 ± 21.5	1579.16 ± 103.81	0.11 ± 0.01	65.32 ± 5.92
Cd_1_+Ca_H_	194.4 ± 21.55	1722.32 ± 25.97	0.11 ± 0.01	68.05 ± 6.50

Data are presented as mean ± SD (*n* = 10). ^a^ Mean Food efficiency = Total weight gain/Total food consumption. The Control, Cd_1_, Cd_1_+Ca_L_, Cd_1_+Ca_M_, and Cd_1_+Ca_H_ groups respectively represent animals fed the AIN93G diet without Cd, with 1 mg Cd/kg, with 1 mg Cd/kg plus 0.15% Ca supplementation, with 1 mg Cd/kg plus 0.4% Ca supplementation, and with 1 mg Cd/kg plus 0.6% Ca supplementation.

**Table 4:** Hematological parameters of female rats after 90 days in the study of Ca supplementation with dietary exposure to 1 mg Cd/Kg (Exp. 1).

Parameter	Units	Control	Cd_1_	Cd_1_+Ca_L_	Cd_1_+Ca_M_	Cd_1_+Ca_H_
HGB	g/L	148.5 ± 5	155 ± 9	152 ± 5	165 ± 15	151 ± 6
RBC	10^12^/L	8.045 ± 0.42	8.08 ± 0.42	8.15 ± 0.36	8.62 ± 0.66	8.41 ± 0.43
MCH	pg	18.3 ± 0.75	19.11 ± 0.63	18.73 ± 0.73	19.1 ± 0.84	18.04 ± 0.59
MCHC	g/L	341 ± 5.42	338.3 ± 5.81	341 ± 3.16	346.8 ± 2.82	348.8 ± 3.74
HCT	%	43.55 ± 1.73	45.68 ± 2.9	44.68 ± 1.16	47.51 ± 4.34	43.4 ± 1.45
MCV	fL	53.67 ± 1.94	56.55 ± 2.2	54.88 ± 2.03	55.12 ± 2.29	51.71 ± 1.85
PLT	10^9^/L	931.1 ± 132	958 ± 166	864 ± 228	870 ± 86	859 ± 88
WBC	10^9^/L	8.125 ± 1.77	8.15 ± 2.31	8.49 ± 2.16	9.71 ± 4.26	12.85 ± 3.74 *
NEUT	10^9^/L	0.927 ± 0.32	1.87 ± 0.94 *	1.39 ± 0.47 *	1.53 ± 0.55 *	1.45 ± 0.5 *
LYM	10^9^/L	6.956 ± 1.73	6.04 ± 2.16	5.82 ± 1.92	7.9 ± 4.04	10.98 ± 3.27 *
MONO	10^9^/L	0.1471 ± 0.07	0.15 ± 0.09	0.15 ± 0.07	0.2 ± 0.18	0.33 ± 0.13 *
EOS	10^9^/L	0.093 ± 0.05	0.09 ± 0.04	0.12 ± 0.09 *	0.07 ± 0.02	0.09 ± 0.05
BASO	10^9^/L	0.002 ± 0	0 ± 0	0 ± 0	0.01 ± 0.01	0.01 ± 0.01
NEUT%	%	11.871 ± 4.53	16.69 ± 6.87	16.78 ± 5.8	17.87 ± 8.79	11.99 ± 2.83
LYM%	%	85.22 ± 5.11	73.51 ± 11.99	79.93 ± 5.42	73.85 ± 24.8	85.33 ± 2.86
MONO%	%	1.88 ± 0.87	1.74 ± 0.71	1.88 ± 0.84	1.92 ± 1.6	2.66 ± 0.81
EOS%	%	1.16 ± 0.52	1.21 ± 0.49	1.55 ± 1.18	0.9 ± 0.41	0.72 ± 0.26
BASO%	%	0.06 ± 0.04	0.05 ± 0.03	0.06 ± 0.04	0.12 ± 0.06	0.1 ± 0.05

Data are presented as mean ± SD (*n* = 10). * *p* < 0.05 by ANOVA and Dunnett’s, vs. control. The Control, Cd_1_, Cd_1_+Ca_L_, Cd_1_+Ca_M_, and Cd_1_+Ca_H_ groups respectively represent animals fed the AIN93G diet without Cd, with 1 mg Cd/kg, with 1 mg Cd/kg plus 0.15% Ca supplementation, with 1 mg Cd/kg plus 0.4% Ca supplementation, and with 1 mg Cd/kg plus 0.6% Ca supplementation. HGB, hemoglobin; RBC, red blood cell count; MCH, mean corpuscular hemoglobin; MCHC, mean corpuscular hemoglobin concentration; HCT, hematocrit; MCV, mean corpuscular volume; PLT, platelet count; WBC, white blood cell count; NEUT, neutrophil count; LYM, lymphocyte count; MONO, monocyte count; EOS, eosinophil count; BASO, basophils.

**Table 5:** Serum chemistry parameters of female rats after 90 days in the study of Ca supplementation with dietary exposure to 1 mg Cd/Kg (Exp. 1).

Parameter	Units	Control	Cd_1_	Cd_1_+ Ca_L_	Cd_1_+Ca_M_	Cd_1_+Ca_H_
AST	IU/L	144 ± 28.42	165.3 ± 42.27	182 ± 103.63	256.3 ± 205.66	137.9 ± 28.59
ALT	IU/L	29.6 ± 6.31	31.4 ± 4.86	37.7 ± 18.66	49.2 ± 31.07	33.6 ± 8.04
Blood urea nitrogen	mmol/L	5.84 ± 2	6.14 ± 0.89	7.06 ± 1.62	8.97 ± 2.1 *^,#^	9.12 ± 3.23 *^,#^
Creatinine	umol/L	41.8 ± 6.94	49.3 ± 9.68	51.5 ± 9.47	52 ± 19.9	45.6 ± 9.88
Total cholesterol	mmol/L	2.2 ± 0.34	2.36 ± 0.51	1.95 ± 0.39	2.4 ± 0.64	2.17 ± 0.38
Triglycerides	mmol/L	0.57 ± 0.14	0.56 ± 0.24	0.5 ± 0.11	0.44 ± 0.15	0.39 ± 0.08
Glucose	mmol/L	12.86 ± 3	11.98 ± 4.3	12.14 ± 2.31	13.23 ± 3.88	10.57 ± 2.55
Total protein	g/L	75.51 ± 4.74	76.97 ± 4.88	71.59 ± 2.46	70.78 ± 4.96 *	71.12 ± 3.23
Albumin	g/L	37.76 ± 2.24	39.12 ± 3.92	36.98 ± 2.02	36.55 ± 2.81	37.08 ± 2.28
Globulin	g/L	37.75 ± 3.46	37.85 ± 3.09	34.71 ± 1.57 *	34.23 ± 2.57 *	34.04 ± 2.2 *
Albumin:Globulin	Ratio	1.01 ± 0.09	1.04 ± 0.13	1.07 ± 0.08	1.07 ± 0.06	1.09 ± 0.09
Calcium	mmol/L	2.66 ± 0.14	2.63 ± 0.12	2.55 ± 0.07	2.72 ± 0.13	2.73 ± 0.12
Phosphorus	mmol/L	2.2 ± 0.64	2.59 ± 0.65	2.75 ± 0.95	3.85 ± 1.37 *	2.88 ± 0.44
Iron	μg/dL	334.6 ± 116.18	224.7 ± 67.9 *	242.64 ± 67.3	312.56 ± 66.1	399.88 ± 38.6
Zinc	μmol/L	25.6 ± 4.67	21.1 ± 2.62 *	20.3 ± 2.20 *	19.6 ± 2.02 *	20.9 ± 3.06 *
Copper	μmol/L	34.9 ± 5.61	29.7 ± 5.93	25.1 ± 3.29 *	28.1 ± 3.97 *	29.6 ± 4.83
1,25-(OH)_2_-VitD_3_	ng/mL	7.43 ± 1.63	7.99 ± 2.06	6.91 ± 0.83	6.90 ± 1.71	8.42 ± 2.67

* *p* < 0.05 by ANOVA and Dunnett’s, vs. control; ^#^ *p* < 0.05 by ANOVA and Dunnett’s, vs. Cd_1_ group. The Control, Cd_1_, Cd_1_+Ca_L_, Cd_1_+Ca_M_, and Cd_1_+Ca_H_ groups respectively represent animals fed with the AIN93G diet without Cd, with 1 mg Cd/kg, with 1 mg Cd/kg plus 0.15% Ca supplementation, with 1 mg Cd/kg plus 0.4% Ca supplementation, and with 1 mg Cd/kg plus 0.6% Ca supplementation. AST = aspartate aminotransferase; ALT = alanine aminotransferase.

**Table 6:** Fe, Zn, Cu concentrations in kidney cortex and liver, and Ca concentration in femur in the 90-day oral toxicity study of Ca supplementation exposure to 1 mg Cd/Kg diet (Exp 1).

Parameter (ug/g)	Control	Cd_1_	Cd_1_+Ca_L_	Cd_1_+Ca_M_	Cd_1_+Ca_H_
Fe content in liver	434.99 ± 99.29	494.44 ± 133.35	397.39 ± 105.18	389.65 ± 56.40	277.38 ± 104.47 *^,#^
Fe content in kidney	467.75 ± 107.84	457.65 ± 110.05	308.41 ± 65.42	302.01 ± 69.40	228.00 ± 48.22 *^,#^
Zn content in liver	40.42 ± 2.88	40.38 ± 3.85	43.00 ± 6.34	39.96 ± 4.88	40.34 ± 2.83
Zn content in kidney	57.56 ± 7.31	57.19 ± 7.16	54.95 ± 3.82	56.42 ± 3.73	57.19 ± 9.27
Cu content in liver	1.09 ± 0.26	0.94 ± 0.16	1.02 ± 0.34	1.19 ± 0.27	0.90 ± 0.08
Cu content in kidney	2.81 ± 0.79	2.46 ± 0.82	2.70 ± 0.60	2.31 ± 0.68	2.11 ± 0.76
Ca content in femur	2503.78 ± 149.36	2492.26 ± 210.59	2486.75 ± 148.93	2470.69 ± 121.19	2361.25 ± 196.84

Data are mean ± SD (*n* = 10). * *p* < 0.05 by ANOVA and Dunnett’s, vs. control; ^#^ *p* < 0.05 by ANOVA and Dunnett’s, vs. Cd_1_ group. The Control, Cd_1_, Cd_1_+Ca_L_, Cd_1_+Ca_M_, and Cd_1_+Ca_H_ groups respectively represent animals fed with the AIN93G diet without Cd, with 1 mg Cd/kg, with 1 mg Cd/kg plus 0.15% Ca supplementation, with 1 mg Cd/kg plus 0.4% Ca supplementation, and with 1 mg Cd/kg plus 0.6% Ca supplementation.

**Table 7:** Body weight of female rats in the study of moderate Ca supplementation exposure to different diet Cd (Exp 2).

Week	Control	1 mg/Kg Cd Diet		5 mg/Kg Cd Diet		50 mg/Kg Cd Diet	
		Ca Supplementation		Ca Supplementation		Ca Supplementation	
		−	+	−	+	−	+
0	127.90 ± 12.92	129.20 ± 16.75	137.60 ± 11.98	132.80 ± 14.46	129.90 ± 12.96	131.50 ± 14.78	134.90 ± 14.67
1	154.20 ± 16.54	155.60 ± 21.98	158.00 ± 13.19	156.20 ± 15.72	150.80 ± 14.82	152.70 ± 16.21	153.00 ± 17.56
2	189.70 ± 21.36	194.20 ± 27.07	191.20 ± 18.62	191.70 ± 16.12	187.00 ± 19.37	187.30 ± 15.49	187.70 ± 19.55
3	226.90 ± 33.38	234.10 ± 38.19	222.40 ± 18.59	224.60 ± 20.60	219.40 ± 23.27	216.40 ± 19.39	217.70 ± 23.09
4	257.00 ± 42.05	264.60 ± 50.24	244.80 ± 24.40	247.60 ± 22.68	245.40 ± 27.12	238.30 ± 16.89	239.10 ± 24.25
5	274.60 ± 43.73	286.40 ± 55.73	262.00 ± 23.76	267.50 ± 19.56	265.20 ± 35.65	255.20 ± 16.42	255.00 ± 28.24
6	280.10 ± 47.27	297.20 ± 61.41	272.40 ± 23.01	280.10 ± 23.85	275.00 ± 29.83	268.30 ± 20.06	263.80 ± 27.00
7	289.00 ± 45.44	309.00 ± 70.81	277.70 ± 28.93	283.00 ± 24.79	280.20 ± 29.27	270.80 ± 22.82	265.50 ± 26.97
8	301.40 ± 48.40	330.80 ± 75.48	291.40 ± 27.26	294.30 ± 24.24	294.20 ± 31.39	281.50 ± 20.11	281.70 ± 28.74
9	306.20 ± 49.64	338.60 ± 82.78	294.30 ± 26.22	297.60 ± 23.74	296.50 ± 30.94	286.20 ± 19.63	281.60 ± 29.17
10	315.00 ± 53.04	352.20 ± 94.78	304.10 ± 27.21	307.60 ± 22.58	305.10 ± 32.50	294.10 ± 18.71	287.40 ± 28.81
11	326.40 ± 54.54	348.70 ± 87.49	308.60 ± 28.05	310.10 ± 23.27	315.80 ± 34.09	303.20 ± 17.49	286.90 ± 28.65
12	323.50 ± 52.83	354.30 ± 91.43	316.70 ± 26.13	303.50 ± 30.00	312.90 ± 36.70	301.50 ± 18.71	291.10 ± 29.71
13	329.40 ± 54.34	359.80 ± 93.95	324.10 ± 29.57	310.30 ± 30.15	308.60 ± 36.55	317.50 ± 20.42	300.40 ± 31.59

Data are mean ± SD (*n* = 10). The Control groups represent animals fed with the AIN93G diet without Cd. −, without Ca supplementation; +, with 0.4% Ca supplementation.

**Table 8:** Cd concentrations in kidney, liver and femurs in the study of moderate Ca supplementation exposure to different diet Cd (Exp 2).

Cd (mg/kg Diet)	Cd Concentrations in Kidney (ug/g)			Cd Concentrations in Liver (ug/g)			Cd Concentrations in Femurs (ug/g)		
	Ca Supplementation		*p*	Ca Supplementation		*p*	Ca Supplementation		*p*
	−	+		−	+		−	+	
0	BDL	--	--	BDL	--	--	BDL	--	--
1	2.64 ± 0.91 *	2.73 ± 0.53	0.79	1.03 ± 0.43 *	0.79 ± 0.4	0.397	0.07 ± 0.01 *	0.07 ± 0.02	0.999
5	14.28 ± 5.30 *	15.57 ± 5.31	0.593	15.36 ± 5.37 *	14.55 ± 6.24	0.759	0.19 ± 0.07 *	0.21 ± 0.11	0.633
50	88.01 ± 7.33 *	88.31 ± 24.71	0.971	52.59 ± 5.52 *	51.81 ± 9.53	0.825	1.04 ± 0.16 *	1.07 ± 0.19	0.701
*p*	<0.001 **			<0.001 **			<0.001 **		

Data are mean ± SD (*n* = 10). ** *p* < 0.05 by ANOVA; * *p* < 0.05 by ANOVA and Dunnett’s, vs. control. BDL, Below Detection Limit; The detection limits were 0.006 μg/g for Cd. **−**, without Ca supplementation; **+**, with 0.4% Ca supplementation.

**Table 9:** Cd concentrations in femur and serum markers in the study of moderate Ca supplementation exposure to different diet Cd (Exp 2).

Cd (mg/kg Diet)	Ca Concentrations in Femur (ug/g)			Serum PINP (ug/L)			Serum β-CTX (ug/L)		
	Ca Supplementation		*p*	Ca Supplementation		*p*	Ca Supplementation		*p*
	−	+		−	+		−	+	
0	2513.78 ± 130.99	--	--	13.74 ± 2.36	--	--	130.92 ± 25.81	--	--
1	2394.48 ± 174.66	2460.69 ± 124.24	0.275	15.72 ± 2.11	14.76 ± 2.8	0.397	129.4 ± 23.29	142.08 ± 18.3	0.192
5	2417.398 ± 185.46	2315.168 ± 125.59	0.165	70 ± 10.13 *	79.7 ± 10.2	0.045 ^#^	140.21 ± 29.67	137.92 ± 21.24	0.845
50	2348.728 ± 140.56 *	2500.518 ± 91.45	0.01 ^#^	15.39 ± 2.75	69.32 ± 12.44	<0.000 ^#^	126.42 ± 14.42	150.31 ± 17.08	0.003 ^#^
*p*	0.135			<0.001 **			0.609		

Data are mean ± SD (*n* = 10). ** *p* < 0.05 by ANOVA; ^#^ *p* < 0.05 by t test; * *p* < 0.05, vs. control. −, without Ca supplementation; +, with 0.4% Ca supplementation. PINP, N-terminal propeptide of type I procollagen; β-CTX, C-terminal telopeptide fragments of the type I collagen.

**Table 10:** Three-point flexural properties of femurs in the study of moderate Ca supplementation exposure to different diet Cd (Exp 2).

**Cd (mg/kg Diet)**	**Young Modulus (MPa)**			**Fracture Stress(MPa)**			**Fracture Load(N)**		
	**Ca Supplementation**		***p***	**Ca Supplementation**		***p***	**Ca Supplementation**		***p***
	**−**	**+**		**−**	**+**		**−**	**+**	
0	1666.59 ± 189.71	--	--	13.82 ± 1.99	--	--	106.84 ± 11.33	--	--
1	1530.59 ± 135.74	1400.98 ± 203.95	0.112	13.08 ± 1.76	12.43 ± 0.84	0.314	109.31 ± 23.63	95.74 ± 8.72	0.116
5	1303.28 ± 139.62 *	1633.92 ± 183.94	<0.001 ^#^	11.73 ± 0.93 *	12.90 ± 1.473	0.048 ^#^	112.09 ± 18.40	104.965 ± 14.34	0.347
50	1243.19 ± 115.23 *	1651.22 ± 120.65	<0.001 ^#^	11.92 ± 1.48 *	13.25 ± 1.27	0.045 ^#^	100.01 ± 6.59	101.48 ± 11.82	0.737
*p*	<0.001 **			0.017 **			0.188		
**Cd (mg/Kg diet)**	**Yield Stress(MPa)**			**Stiffness(N/mm)**			**Yield Load(N)**		
	**Ca Supplementation**		***p***	**Ca Supplementation**		***p***	**Ca Supplementation**		***p***
	**−**	**+**		**−**	**+**		**−**	**+**	
0	12.02 ± 1.79	--	--	223.27 ± 37.18	--	--	92.88 ± 9.23	--	--
1	10.92 ± 1.44	11.41 ± 1.35	0.447	224.25 ± 53.50	209.71 ± 19.34	0.436	89.832 ± 11.10	87.78 ± 8.03	0.642
5	9.60 ± 1.52 *	11.14 ± 2.33	0.097	256.86 ± 40.45	262.34 ± 51.22	0.794	100.56 ± 9.98	102.00 ± 11.97	0.774
50	10.20 ± 1.57 *	10.63 ± 1.97	0.599	207.38 ± 18.71	201.011 ± 19.67	0.467	88.30 ± 9.41	87.30 ± 10.05	0.819
*p*	0.011 **			0.017 **			0.043 **		

Data are mean ± SD (*n* = 10). ** *p* < 0.05 by ANOVA and Dunnett’s; ^#^ *p* < 0.05 by t test; * *p* < 0.05, vs. control. −, without Ca supplementation; +, with 0.4% Ca supplementation.

**Table 11:** Expression changes of osteogenic formation in the femurs using quantitative real-time polymerase chain reaction in the study of moderate Ca supplementation exposure to different diet Cd (Exp 2).

**Cd (mg/kg Diet)**	**Dmp1 (Fold Change)**			**Col1a1 (Fold Change)**			**Sost (Fold Change)**		
	**Ca Supplementation**		***p***	**Ca Supplementation**		***p***	**Ca Supplementation**		***p***
	**−**	**+**		**−**	**+**		**−**	**+**	
0	1 ± 0.21	--	--	1 ± 0.55	--	--	1 ± 0.2	--	--
1	0.788 ± 0.11	1.90 ± 0.1	<0.001 ^#^	3.67 ± 0.04	13.07 ± 0.36	<0.001 ^#^	0.9 ± 0.11	1.24 ± 0.2	<0.001 ^#^
5	3.04 ± 0.43 *	3.58 ± 0.42	0.01 ^#^	8.26 ± 0.68 *	8.56 ± 0.27	0.21	0.66 ± 0.49	0.84 ± 0.26	0.318
50	1.59 ± 0.31	2.28 ± 0.16	<0.001 ^#^	2.62 ± 0.21	5.44 ± 1.15	<0.001 ^#^	0.88 ± 0.25	1.04 ± 0.15	0.099
*p*	<0.001 **			<0.001 **			0.092		
**Cd (mg/kg Diet)**	**Runx2 (Fold Change)**			**ALP (Fold Change)**			**Phex (Fold Change)**		
	**Ca Supplementation**		***p***	**Ca Supplementation**		***p***	**Ca Supplementation**		***p***
	**−**	**+**		**−**	**+**		**−**	**+**	
0	1 ± 0.07	--	--	1 ± 0.09	--	--	1 ± 0.14	--	--
1	1.11 ± 0.42	3.42 ± 0.63	<0.001 ^#^	3040.18 ± 0.36 *	3878.29 ± 0.26	<0.001 ^#^	1.03 ± 0.11	0.73 ± 0.09	<0.001 ^#^
5	7.02 ± 0.85 *	2.14 ± 0.07	<0.001 ^#^	3388.752 ± 0.66 *	479.094 ± 0.04	<0.001 ^#^	1.01 ± 0.16	1.16 ± 0.23	0.107
50	4.11 ± 0.18 *	2.6 ± 0.1	<0.001 ^#^	297.03 ± 0.1 *	94.56 ± 0.09	<0.001 ^#^	1.01 ± 0.07	1.05 ± 0.09	0.281
*p*	<0.001 **			<0.001 **			0.958		
**Cd (mg/kg Diet)**	**Osterix (Fold Change)**			**OCN (Fold Change)**			**OPN (Fold Change)**		
	**Ca Supplementation**		***p***	**Ca Supplementation**		***p***	**Ca Supplementation**		***p***
	**−**	**+**		**−**	**+**		**−**	**+**	
0	1 ± 0.09	--	--	1 ± 0.13	--	--	1 ± 0.12	--	--
1	0.92 ± 0.15	1.22 ± 0.13	<0.001 ^#^	0.64 ± 0.53 *	0.52 ± 0.16	0.502	0.75 ± 0.10	0.77 ± 0.43	0.888
5	0.79 ± 0.38	1.23 ± 0.36	0.016 ^#^	2.90 ± 0.18 *	0.66 ± 0.13	<0.001 ^#^	1.19 ± 0.49	1.23 ± 0.19	0.812
50	0.55 ± 0.12 *	0.59 ± 0.12	0.465	1.32 ± 0.16	1.28 ± 0.13	0.546	1.22 ± 0.12 *	1.33 ± 0.18	0.124
*p*	<0.001 **			<0.001 **			0.001 **		

Data are expressed as fold changes (mean ±SD, *n* = 10), normalized to GAPDH mRNA expression, where the values for the control mice were set at 1.0. Fold change = 2^−ΔΔCt^; ΔCt = Ct_target gene_−Ct_GAPDH_; ΔΔCt = ΔCt_targe group_−ΔCt_control_. ** *p* < 0.05 by ANOVA and Dunnett’s; ^#^ *p* < 0.05 by t test; * *p* < 0.05, vs. control. Dmp1, dentin matrix protein 1; Col1a1, collagen type 1; Sost, sclerostin; Runx2, runt-domain transcription factor 2; ALP, alkaline phosphatase; Phex, phosphate-regulating gene with homologies to endopeptidases on the X-chromosome; OCN, osteocalcin; OPN, osteopontin.

**Table 12:** Serum calciotropic hormones in the study of moderate Ca supplementation exposure to different diet Cd (Exp 2).

Cd (mg/kg Diet)	1,25-(OH)_2_D_3_ (ng/L)			Klotho (ng/L)			Intact FGF23 (ng/L)		
	Ca Supplementation		*p*	Ca Supplementation		*p*	Ca Supplementation		*p*
	−	+		−	+		−	+	
0	62.96 ± 4.93	--	--	3.82 ± 1.63	--	--	955.23 ± 127.44	--	--
1	78 ± 11.16 *	69.79 ± 10.72	0.11	3.45 ± 1.09	2.66 ± 0.48	0.048 ^#^	889.725 ± 139.009	934.57 ± 178.73	0.539
5	61.75 ± 6.91	65.65 ± 8	0.258	3.24 ± 0.55	2.15 ± 0.25	<0.001 ^#^	950.145 ± 169.11	1004.227 ± 132.27	0.435
50	66.12 ± 12.23	72.13 ± 10.33	0.25	3.34 ± 0.89	2.63 ± 0.56	0.046 ^#^	955.05 ± 153.40	970.53 ± 154.145	0.824
*p*	0.001 **			0.715			0.708		

Data are mean ± SD (*n* = 10). ** *p* < 0.05 by ANOVA and Dunnett’s; # *p* < 0.05 by t test; * *p* < 0.05, vs. control. −, without Ca supplementation; +, with 0.4% Ca supplementation. FGF23, fibroblast growth factor 23.

**Table 13:** Expression changes of FGF23/Klotho-associated gene using quantitative real-time polymerase chain reaction in the kidney in the study of moderate Ca supplementation exposure to different diet Cd (Exp 2).

**Cd (mg/kg Diet)**	**Klotho (Fold Change)**			**Fgfr1 (Fold Change)**			**Napi2a (Fold Change)**		
	**Ca Supplementation**		***p***	**Ca Supplementation**		***p***	**Ca Supplementation**		***p***
	**−**	**+**		**−**	**+**		**−**	**+**	
0	1.00 ± 0.10	--	--	1.00 ± 0.20	--	--	1.00 ± 0.25	--	--
1	2.07 ± 0.77 *	0.54 ± 0.14	<0.001 ^#^	2.07 ± 1.32	1.51 ± 0.90	0.282	4.44 ± 2.89 *	1.31 ± 1.21	0.008 ^#^
5	24.64 ± 9.07 *	21.88 ± 6.89	0.453 ^#^	1.90 ± 0.93	2.84 ± 0.89	0.032 ^#^	2.82 ± 1.15 *	1.52 ± 0.83	0.009 ^#^
50	6.91 ± 1.01 *	3.28 ± 1.09	<0.001 ^#^	2.94 ± 0.85 *	4.04 ± 1.43	0.05 ^#^	2.00 ± 0.95 *	1.17 ± 0.68	0.037^#^
*p*	<0.001 **			0.001 **			<0.001 **		
**Cd (mg/kg Diet)**	**Cyp24a1 (Fold Change)**			**Cyp27b1 (Fold Change)**					
	**Ca Supplementation**		***p***	**Ca Supplementation**		***p***			
	**−**	**+**		**−**	**+**				
0	1.00 ± 0.10	--	--	1.00 ± 0.34	--	--			
1	2.11 ± 1.57 *	1.23 ± 1.04	0.156	1.61 ± 0.87 *	11.99 ± 3.79	<0.001 ^#^			
5	0.57 ± 0.43 *	0.72 ± 0.48	0.471	0.52 ± 0.82 *	0.83 ± 0.49	0.318			
50	0.36 ± 0.21 *	1.76 ± 1.36	0.01^#^	0.44 ± 0.33 *	4.80 ± 1.31	<0.001 ^#^			
*p*	<0.001 **			0.001 **					

Data are expressed as fold changes (mean ± SD, *n* = 10), normalized to GAPDH mRNA expression, where the values for the control mice were set at 1.0. Fold change = 2^−ΔΔCt^; ΔCt = Ct_target gene_−Ct_GAPDH_; ΔΔCt = ΔCt_targe group_−ΔCt_control_. Data are mean ± SD (*n* = 10). ** *p* < 0.05 by ANOVA; # *p* < 0.05 by t test; * *p* < 0.05, vs. Fgfr1, fibroblast growth factor receptor 1.

**Table 14:** Expression changes of FGF23/Klotho-associated gene using quantitative real-time polymerase chain reaction in the femurs in the study of moderate Ca supplementation exposure to different diet Cd (Exp 2).

**Cd (mg/kg Diet)**	**OPG (Fold Change)**			**RANKL (Fold Change)**			**Axin2 (Fold Change)**		
	**Ca Supplementation**		***p***	**Ca Supplementation**		***p***	**Ca Supplementation**		***p***
	**−**	**+**		**−**	**+**		**−**	**+**	
0	1.00 ± 0.05	--	--	1.00 ± 0.14	--	--	1.00 ± 0.09	--	--
1	11.57 ± 0.18 *	18.04 ± 0.45	<0.001 ^#^	0.91 ± 0.15	0.88 ± 0.20	0.709	5.93 ± 0.20 *	8.89 ± 0.14	<0.001 ^#^
5	7.79 ± 0.12 *	15.45 ± 0.57	<0.001 ^#^	0.92 ± 0.10	1.00 ± 0.13	0.14	5.65 ± 0.13 *	3.75 ± 0.04	<0.001 ^#^
50	17.62 ± 0.10 *	35.66 ± 0.25	<0.001 ^#^	0.97 ± 0.07	0.93 ± 0.04	0.133	10.20 ± 0.17 *	16.33 ± 0.03	<0.001 ^#^
*p*	<0.001 **			0.302			<0.001 **		
**Cd (mg/kg Diet)**	**FGF23 (Fold Change)**			**Tcf1 (Fold Change)**			**Dkk1 (Fold Change)**		
	**Ca Supplementation**		***p***	**Ca Supplementation**		***p***	**Ca Supplementation**		***p***
	**−**	**+**		**−**	**+**		**−**	**+**	
0	1.00 ± 0.05	--	--	1.00 ± 0.08	--	--	1.00 ± 0.15	--	--
1	4.17 ± 0.19 *	13.97 ± 0.86	<0.001 ^#^	0.88 ± 0.08 *	0.72 ± 0.16	0.01 ^#^	3.68 ± 0.14 *	3.08 ± 0.03	<0.001 ^#^
5	2.55 ± 0.34 *	4.84 ± 0.10	<0.001 ^#^	0.49 ± 0.17 *	0.62 ± 0.32	0.27	7.74 ± 0.33 *	1.23 ± 0.14	<0.001 ^#^
50	9.39 ± 0.42 *	16.23 ± 0.15	<0.001 ^#^	0.45 ± 0.12 *	0.60 ± 0.15	0.04 ^#^	6.87 ± 0.12 *	25.22 ± 0.25	<0.001 ^#^
*p*	<0.001 **			<0.001 **			<0.001 **		
**Cd (mg/kg Diet)**	**Wnt1 (Fold Change)**			**β-catenin (Fold Change)**					
	**Ca supplementation**		***p***	**Ca Supplementation**		***p***			
	**−**	**+**		**−**	**+**				
0	1.00 ± 0.37	——	——	1.00 ± 0.21	——	——			
1	0.41 ± 0.14 *	0.29 ± 0.13	0.062	1.34 ± 0.52 *	1.46 ± 0.09	0.481			
5	1.71 ± 0.42 *	1.87 ± 0.83	0.593	4.00 ± 0.20 *	3.43 ± 0.74	0.03 ^#^			
50	0.42 ± 0.15 *	0.33 ± 0.06	0.094	12.13 ± 1.30 *	9.23 ± 0.15	<0.001 ^#^			
*p*	<0.001 **			0.001 **					

Data are expressed as fold changes (mean ± SD, *n* = 10), normalized to GAPDH mRNA expression, where the values for the control mice were set at 1.0. Fold change = 2^-ΔΔCt^; ΔCt = Ct_target gene_−Ct_GAPDH_; ΔΔCt = ΔCt_targe group_−ΔCt_control_. ** *p* < 0.05 by ANOVA; ^#^ *p* < 0.05 by t test; * *p* < 0.05, vs. control. Fgfr1, fibroblast growth factor receptor 1; OPG, osteoprotegerin; RANKL, receptor activator of nuclear factor-kappa B ligand-osteoprotegerin; Dkk1, Dickkopf-1; FGF23, fibroblast growth factor 23.

## Supplementary Materials

The following are available online at https://www.mdpi.com/2072-6643/11/4/849/s1, Figure S1: Serum C-terminal FGF23 in the study of moderate Ca supplementation exposure to different dietary levels of Cd, Table S1: Composition of AIN-93G diets (g/kg), Table S2: Primer sequences for quantitative real-time polymerase chain reaction, Table S3: Summary of food consumption data (g/week) in the 90-day oral toxicity study (mean ± SD, *n* = 10), Table S4: Absolute organ weight and relative organ weight in the 90-day oral toxicity study (mean ± SD, *n* = 10), Table S5: Summary of histopathology of the kidneys in the 90-day oral toxicity study (*n* = 10), Table S6: Left femur dry weight in experiment 2 (g, *n* = 10).
