# Supplementary material for: Protective Effects of Moderate Ca Supplementation against Cd-Induced Bone Damage under Different Population-Relevant Doses in Young Female Rats
Source: Nutrients. 2019 Apr 15;11(4):849. doi: 10.3390/nu11040849 (PMC6521033; doi:10.3390/nu11040849)
Supplement: Supplementary file 1 [file nutrients-11-00849-s001.pdf]

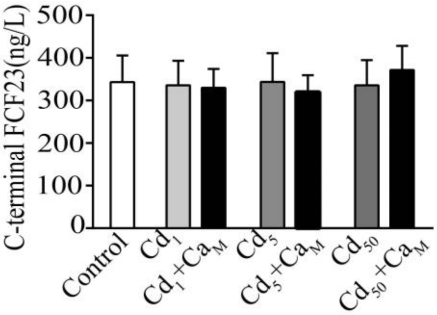


Figure S1: Serum C-terminal FCF23 in the study of moderate Ca supplementation exposure to different diet Cd.

Supplementary Table S1 Composition of AIN-93G diets (g/kg)

| Ingredient (g/kg) | |
|---|---|
| Casein | 200 |
| Cornstarch | 397.486 |
| Dextrin | 132 |
| Sucrose | 100 |
| Soybean oil | 70 |
| Cellulose | 50 |
| Mineral mix[a] | 35 |
| Vitamin mix[b] | 10 |
| L-Cystine | 3 |
| Choline bitartrate | 2.5 |
| Tert-Butylhydroquinone | 0.014 |

a The composition of mineral mixture was based on AIN-93G. It provided (mg/kg diet): Ca, 5000; P,3000; Mg, 513; Fe, 45; Zn, 38 and Cu, 6.

b The composition of vitamin mixture was based on AIN-93. The diets contain 1 IU Vitamin D3 per 1g diet

Supplementary Table S2 Primer sequences for quantitative real-time polymerase chain reaction.

| | Genes | primers |
|---|---|---|
| NM_017008.4 | R-GAPDH-S | CTGGAGAAACCTGCCAAGTATG |
| | R-GAPDH-A | GGTGGAAGAATGGGAGTTGCT |
| NM_031336.1 | R-Klotho -S | TCCGACGAGGACTCTTCTATGT |
| | R- Klotho -A | GGCTACAACCCCGTCTACTTTA |
| NM_024146.1 | R- Fgfr1-S | GGAGGCTACAAGGTTCGTTACG |
| | R- Fgfr1-A | AATGCTCCCATACTCGTTCTCC |
| NM_053763.1 | R-Cyp27b1-S | CGGGACACTTCGCACAGTTTAT |
| | R-Cyp27b1-A | TTCTTCACCATCCGCCGTTAG |
| NM_013030.1 | R-Napi2a-S | TCACAGTCTCATTCGGATTTGGT |
| | R-Napi2a-A | TGACGAGGAGGATAAGACAAGTGC |
| NM_201635.3 | R-Cyp24a1-S | AATGAGGTCTTGGCTGACTTTCT |

| | | |
|---|---|---|
| | R-Cyp24a1-A | TCACCATCATCTTCCCAAACG |
| NM_053304.1 | R-Colla1(RZ）-S | AGAGGCATAAAGGGTCATCGTG |
| | R-Colla1(RZ)-A | AGACCGTTGAGTCCATCTTTGC |
| NM_013004.1 | R-Phex-S | CCAAACCACTGCTCCACATCTT |
| | R-Phex-A | TATGCTCATTGGATGCCTTGTC |
| NM_001278483.1 | R-Runx2(RZ)-S | GCGGACGAGGCAAGAGTTTC |
| | R-Runx2(RZ)-A | GACTTGGTGCTGAGTTCAGGGAG |
| NM_053650.1 | R-ALP（RZ）-S | GGATCAAAGCAGCATCTTACCAG |
| | R-ALP（RZ）-A | GCTTTCCCATCTTCCGACACT |
| NM_012881.2 | R-OPN(rz)-S | GATGAACAGTATCCCGATGCCA |
| | R-OPN(rz)-A | GTCTTCCCGTTGCTGTCCTGA |
| NM_001037632 | R- Osterix-S | CTGGGAAAAGGAGGCACAAAGA |
| | R- Osterix-A | GGGGAAAGGGTGGGTAGTCATT |
| NM_013414.1 | R- OCN（RZ）-S | AGGGCAGTAAGGTGGTGAATAGA |
| | R- OCN（RZ）-A | GAAGCCAATGTGGTCCGCTA |
| NM_203493.3 | R-Dmp1(RZ)-S | CGCCCATGGCAAATAGTGAC |
| | R- Dmp1 (RZ)-A | CTCCTTATCGGCGTCCATCC |
| NM_130754.1 | R-FGF23(RZ)-S | AGAGCCTATTCAGACACTTCCCC |
| | R-FGF23(RZ)-A | AGCCAGCATCCTCTGATGTGAT |
| NM_030584.1 | R-Sost(RZ)-S | CAACCAGACCATGAACCGGG |
| | R-Sost(RZ)-A | TCACGAAGCGGGTGTAGTG |
| NM_001106350.1 | R-Dkk1-S | GTGTTCTGGTGGACAGTTTGCC |
| | R-Dkk1-A | TCTCAATCTCGTCTGGGCTGAT |
| NM_012870.2 | R-OPG-S | GTGTTCTGGTGGACAGTTTGCC |
| | R-OPG-A | TCTCAATCTCGTCTGGGCTGAT |
| NM_057149.1 | R- RANKL -S | CATCGGGTTCCCATAAAGTCAGT |
| | R- RANKL -A | GCAAATGTTGGCGTACAGGTAAT |
| NM_012669.1 | R-Tcf1-S | CAGCGGAGGTCCCTTAGTCA |
| | R-Tcf1-A | CAGACCAATGACCAGGGTAGAG |
| NM_024355.1 | R-Axin2(RZ)-S | ATGTCCATGACAGACAGTAGCGTAG |
| | R-Axin2(RZ)-A | TGGGTTCTCGGGAAGTGAGG |
| NM_001105714.1 | R-Wnt1(rz)-S | TGGGTTTCTGCTACGTTGCTACT |
| | R-Wnt1(rz)-A | CAGCCTCGGTTGACGATCTT |
| NM_053357.2 | R-β-catenin-S | TGGTGAAAATGCTTGGGTCG |
| | R-β-catenin-A | TCTGAAGGCAGTCTGTCGTAATAG |

**Supplementary Table S3 Summary of food consumption data (g/week) in the 90-day oral toxicity study (mean ± SD, n =10).**

| | **Control** | **$Cd_1$** | **$Cd_1$+ $Ca_L$** | **$Cd_1$+$Ca_M$** | **$Cd_1$+$Ca_H$** |
|---|---|---|---|---|---|
| Week1(day1-7) | 111.34±8.67 | 118.41±2.03 | 104.44±1.48 | 111.62±3.5 | 119.88±3.14 |
| Week2(day8-14) | 111.24±23.59 | 128.02±19.35 | 109.62±29.29 | 114.3±15.94 | 133.06±16.29 |
| Week3(day15-21) | 135.98±7.17 | 144.61±14.52 | 110.58±7.95* | 129.37±9.09 | 148.2±14.62 |
| Week4(day22-28) | 136.64±0.3 | 139.86±7.82 | 127.89±1.84* | 124.21±6.53 | 141.02±9.85 |
| Week5(day29-35) | 124.81±2.8 | 136.26±12.14 | 118.76±0.85* | 117.67±7.53 | 132.44±3.17 |
| Week6(day36-42) | 120.54±3.25 | 141.93±9.48* | 121.38±2.8 | 123.62±8.19 | 132.41±9.41* |
| Week7(day43-49) | 123.79±10.04 | 138.11±22.81 | 116.52±15.05 | 120.06±13.51 | 120.77±17.83 |
| Week8(day50-56) | 123.93±10.88 | 141.4±15.05 | 123.48±5.53 | 126.95±6.6 | 131.39±1.25 |
| Week9(day57-63) | 134.82±14.46 | 137.94±9.04 | 123.3±0.18 | 126.42±4.43 | 130.9±1.03 |
| Week10(d | 126.95±4 | 134.51±2 | 123.09±5 | 125.9±2. | 130.38± |

| | | | | | |
|---|---|---|---|---|---|
| ay64-70) | .76 | .99 | .94* | 25 | 3.36 |
| Week11(day71-77) | 129.47±24.15 | 122.03±18.6 | 120.38±22.41 | 127.55±23.95 | 129.64±23.85 |
| Week12(day78-84) | 113.12±20.22 | 102±0.59 | 133.39±8.45 | 123.03±23.8 | 124.66±19.74 |
| Week13(day85-90) | 101.75±17.8 | 99.6±0.51 | 107.63±7.49 | 108.48±21 | 107.59±17.42 |

* Statistically significant from control, $p<0.05$.

**Supplementary Table S4 Absolute organ weight and relative organ weight in the 90-day oral toxicity study (mean ± SD, n = 10).**

| | Control | $Cd_1$ | $Cd_1$+ $Ca_L$ | $Cd_1$+ $Ca_M$ | $Cd_1$+ $Ca_H$ |
|---|---|---|---|---|---|
| Body weight[a] (g) | 308±52 | 338±59 | 310±28 | 299±27 | 315±30 |
| Heart(g) | 1.01±0.1 | 0.98±0.2 | 0.96±0.19 | 0.92±0.18 | 0.97±0.23 |
| Liver | 8.77±1.24 | 9.18±2.25 | 8.11±0.77 | 7.96±0.79 | 7.89±0.73 |
| Spleen | 0.5±0.12 | 0.53±0.09 | 0.54±0.13 | 0.51±0.14 | 0.48±0.1 |
| Kidney(paired) | 2.14±0.4 | 2.05±0.42 | 1.88±0.23 | 1.88±0.15 | 2.03±0.28 |

| | | | | | |
|---|---|---|---|---|---|
| Uterus | 0.55±0.24 | 0.55±0.16 | 0.57±0.29 | 0.64±0.39 | 0.63±0.17 |
| Ovary(paired) | 0.13±0.05 | 0.2±0.19 | 0.13±0.02 | 0.14±0.02 | 0.13±0.02 |
| Heart/body weight ratios(mg/100 g) | 0.33±0.05 | 0.3±0.06 | 0.31±0.04 | 0.31±0.04 | 0.31±0.06 |
| Liver/body weight ratios(mg/100 g) | 2.88±0.46 | 2.74±0.4 | 2.62±0.22 | 2.65±0.06 | 2.51±0.25 |
| Spleen/body weight ratios(mg/100 g) | 0.16±0.03 | 0.16±0.03 | 0.17±0.04 | 0.17±0.05 | 0.15±0.03 |
| Kidney/body weight ratios(mg/100 g) | 0.7±0.12 | 0.61±0.08 | 0.6±0.05 | 0.63±0.06 | 0.64±0.09 |
| Uterus/body weight ratios(mg/100 g) | 0.19±0.1 | 0.17±0.07 | 0.18±0.08 | 0.21±0.11 | 0.2±0.05 |
| Ovary/body weight ratios(mg/100 g) | 0.04±0.02 | 0.06±0.07 | 0.04±0.01 | 0.05±0.01 | 0.04±0.01 |

[a] Terminal body weight.

**Supplementary Table S5 Summary of histopathology in kidney in the 90-day oral toxicity study ($n$ = 10).**

| | Severity | Control | $Cd_1$ | $Cd_1$+ $Ca_L$ | $Cd_1$+ $Ca_M$ | $Cd_1$+ $Ca_H$ |
|---|---|---|---|---|---|---|
| Tubular vacuolation | ± | 10 | 10 | 9 | 7 | 7 |
| | + | 0 | 0 | 1 | 3 | 3 |

| | | | | | | |
|---|---|---|---|---|---|---|
| Protein cast | ± | 7 | 7 | 4 | 3 | 0 |
| | + | 3 | 3 | 6 | 7 | 10 |
| Calcification | ± | 2 | 2 | 0 | 0 | 0 |
| | + | 8 | 6 | 9 | 5 | 4 |
| | 2+ | 0 | 2 | 1 | 3 | 4 |
| | 3+ | 0 | 0 | 0 | 2 | 2 |
| Interstitial lymphocytic infiltrates | 0 | 0 | 0 | 0 | 2 | 0 |

Severity scale: ±, Minimal; +, Slight; 2+, Moderate; 3+, Marked

**Supplementary Table S6 Left femur dry weight in experiment 2 (g, n = 10).**

| Control | $Cd_1$ | $Cd_1$+ $Ca_M$ | $Cd_5$ | $Cd_5$+ $Ca_M$ | $Cd_{50}$ | $Cd_{50}$+ $Ca_M$ |
|---|---|---|---|---|---|---|
| 0.63±0.06 | 0.69±0.07 | 0.66±0.04 | 0.66±0.07 | 0.68±0.08 | 0.62±0.04 | 0.62±0.07 |
